# Possibility for Transcriptional Targeting of Cancer-Associated Fibroblasts—Limitations and Opportunities

**DOI:** 10.3390/ijms22073298

**Published:** 2021-03-24

**Authors:** Dina V. Antonova, Marina V. Zinovyeva, Liya G. Kondratyeva, Alexander V. Sass, Irina V. Alekseenko, Victor V. Pleshkan

**Affiliations:** 1 Department of Genomics and Postgenomic Technologies, Gene Immunooncotherapy Group, Shemyakin-Ovchinnikov Institute of Bioorganic Chemistry RAS, 117997 Moscow, Russia; tyulkina.dina@mail.ru (D.V.A.); mzinov@mail.ru (M.V.Z.); liakondratyeva@yandex.ru (L.G.K.); avsass@mail.ru (A.V.S.); irina.alekseenko@mail.ru (I.V.A.); 2 Gene Oncotherapy Sector, Institute of Molecular Genetics of National Research Centre “Kurchatov Institute”, 123182 Moscow, Russia; 3 Institute of Oncogynecology and Mammology, National Medical Research Center for Obstetrics, Gynecology and Perinatology named after Academician V.I. Kulakov of the Ministry of Healthcare of Russian Federation, 117997 Moscow, Russia

**Keywords:** transcriptional targeting, promoter, tumor microenvironment, fibroblasts, gene therapy

## Abstract

Cancer-associated fibroblasts (CAF) are attractive therapeutic targets in the tumor microenvironment. The possibility of using CAFs as a source of therapeutic molecules is a challenging approach in gene therapy. This requires transcriptional targeting of transgene expression by cis-regulatory elements (CRE). Little is known about which CREs can provide selective transgene expression in CAFs. We hypothesized that the promoters of *FAP*, *CXCL12*, *IGFBP2*, *CTGF*, *JAG1*, *SNAI1*, and *SPARC* genes, the expression of whose is increased in CAFs, could be used for transcriptional targeting. Analysis of the transcription of the corresponding genes revealed that unique transcription in model CAFs was characteristic for the *CXCL12* and *FAP* genes. However, none of the promoters in luciferase reporter constructs show selective activity in these fibroblasts. The CTGF, IGFBP2, JAG1, and SPARC promoters can provide higher transgene expression in fibroblasts than in cancer cells, but the nonspecific viral promoters CMV, SV40, and the recently studied universal PCNA promoter have the same features. The patterns of changes in activity of various promoters relative to each other observed for human cell lines were similar to the patterns of activity for the same promoters both in vivo and in vitro in mouse models. Our results reveal restrictions and features for CAF transcriptional targeting.

## 1. Introduction

Tumors represent the assembly of cancer and tumor microenvironment cells (TME). Cancer-associated fibroblasts (CAFs) are prominent cells within the tumor microenvironment and are critically involved in cancer progression. Recently, CAFs have emerged as attractive therapeutic targets for gene therapy [1,2,3]. However, targeting CAFs has faced numerous obstacles and challenges. The heterogeneity of specific cell surface markers for CAFs restrains the direct impact on them and it is difficult to target them precisely.

After the successful registration of the gene therapy anticancer drug Imlygic by the Food and Drug Administration (FDA), a huge amount of works in the field of anticancer gene therapy has been published. Currently, great interest in the development of antitumor gene therapy is, where possible, the use of local (intratumoral) drug administration, which reduces the systemic toxicity of drugs [4,5]. By their nature, non-viral vectors are well suited for local administration [6,7]. The production of such drugs in general is cheaper and easier than the production of viral vectors. In addition, such vectors may be safer, although they are inferior in transfection efficiency to viral vectors.

When local administration of the gene therapy drug is used, a variety of tumor cells might be affected—cancer cells and microenvironment cells including CAFs. Cancer cells are well known for their cellular plasticity, contributing to tumor heterogeneity and therapy resistance [8]. This can lead to rapid inactivation of the expression of transgenes. At the same time, the cancer-associated fibroblasts are genetically more stable and have a relatively low cell division rate, thus theoretically they can provide a longer expression. The previously discussed possibility of transcriptional control of the expression of therapeutic genes by using specific regulatory elements (promoters) [9,10] suggests that the use of fibroblast-specific regulatory elements could promote targeted expression of therapeutic genes mainly by cancer-associated fibroblasts.

In this study, we investigated the possibility for transcriptional targeting of cancer-associated fibroblasts. Herein, the promoters of certain genes, the expression of whose were increased in CAFs, were used to evaluate their ability for efficient and selective expression of transgenes in these cells. The analysis of transfected cell populations in a tumor in vivo is quite difficult and elusive. Therefore, cancer cell lines of pancreatic adenocarcinoma, which is characterized by the most developed tumor microenvironment, were chosen as a model for in vitro experiments. A primary culture of human fibroblasts was used as CAFs. Such an approach, although it has several limitations, give us the opportunity to examine in a simple model the possibility of using the promoters of the genes with a high level of expression in the stroma (*FAP*, *CXCL12*, *IGFBP2*, *CTGF*, *JAG1*, *SNAI1*, *SPARC*) as elements for transcriptional targeting of gene expression in tumor fibroblasts.

## 2. Results

### 2.1. Experiment Design

#### 2.1.1. The Choice of the Cell Lines for a Model System

Pancreatic cancer is a tumor with an extremely heterogeneous origin of cancer cells, the sources of which can be neoplastic acinar, ductal, and other cells of the pancreas that have undergone malignant transformation [11,12]. In addition to the cancer cells, the stroma of tumor can expand by 50% [13]. In pancreatic carcinomas, CAFs are the most prominent stroma cell type [14].

The human pancreatic cancer cell lines PANC-1 and MIA PaCa-2 were selected, which are characterized by high expression of mesenchymal markers and which are characterized as highly differentiated cancer cells [15], and AsPC-1 cell line representing low differentiated adenocarcinoma [16]. A primary cell culture obtained from the stroma of a patient with pancreatic cancer, IVP-9TS, was used as a model of cancer-associated fibroblasts [17]. The human lung cancer epithelial cell line Calu-1 was used as a control to assess the possible tissue selectivity of potential stroma CREs.

The CMT 167 lung cancer and the NIH/3T3 fibroblasts were used as mouse cell lines. For in vivo experiments, a syngeneic mouse model of lung carcinoma CMT 167 with subcutaneous inoculation in C57BL mice was chosen.

#### 2.1.2. The Choice of Genes for the Study

We have suggested that the promoters of genes, the expression of whose is increased in the tumor stroma, could be active mainly in the cells of the tumor microenvironment. To test this hypothesis, the following genes *FAP*, *CXCL12*, *IGFBP2*, *CTGF*, *JAG1*, *SNAI1*, and *SPARC* with increased expression in the TME of pancreatic cancer were chosen. Our choice was based on the literature analysis and the previous study of our laboratory on the analysis of transcriptomes of cells of the stroma microenvironment of pancreatic adenocarcinoma by the SAGE (Serial Analysis of Gene Expression) method (Kopantzev, unpublished).

According to the literature, the expression of the *FAP*, *CXCL12*, *CTGF*, and *SNAI1* genes is characteristic of CAFs in the TME of pancreatic cancer [13,18,19,20]. It is known that the *FAP* gene is selectively expressed in stroma fibroblasts of epithelial tumors. FAP is robust and selective marker for reactive mesenchymal stroma cells associated with pathophysiologic tissue remodeling [21]. Due to this high specificity, the FAP protein could be a promising target for anticancer therapy [22]. The *CXCL12* gene encodes a chemokine that is involved in inflammatory processes associated with cell migration. In pancreatic cancer, the CXCL12 is predominantly secreted by CAFs, while its expression in cancer cells is virtually absent [13]. The *CTGF* gene is predominantly expressed by TME fibroblasts [18]. CTGF protein stimulates autophagy, glycolysis, and aging of CAFs, as well as promotes the growth of tumor cells [23]. The *SNAI1* gene encodes the SNAIL transcription factor, which regulates the epithelial-mesenchymal transition in the processes of embryonic development, tissue regeneration, and carcinogenesis. Expression of SNAIL in pancreatic cancer is observed mainly in the nuclei of stroma cells, while it is rarely found in cancer cells [19]. SNAIL is thought to be a marker of activated fibroblasts of the tumor stroma [20].

Expression of the *SPARC* gene, which encodes a protein involved in the remodeling of the extracellular matrix in pancreatic cancer, is characteristic of both cancer cells and CAFs, but in the latter, its expression is much higher [13]. *IGFBP2* gene is a potential stroma-associated biomarker in pancreatic cancer [24]. The *JAG1* gene encodes the Jagged-1 protein, one of ligands that interacts with its respective Notch receptor. The activation of ligand-induced Notch signaling pathway in the CAFs is associated with poor prognosis, metastasis, and aggressiveness of the tumor [25,26,27].

The selected panel of genes, to a certain extent, reflects the characteristic pattern of gene expression of the pancreatic adenocarcinoma microenvironment, with a predominance of genes associated with extracellular matrix remodeling, angiogenesis, metastasis, and growth factors [28,29].

### 2.2. Determination of Transcription Level of the Studied Genes

**Figure 1:**
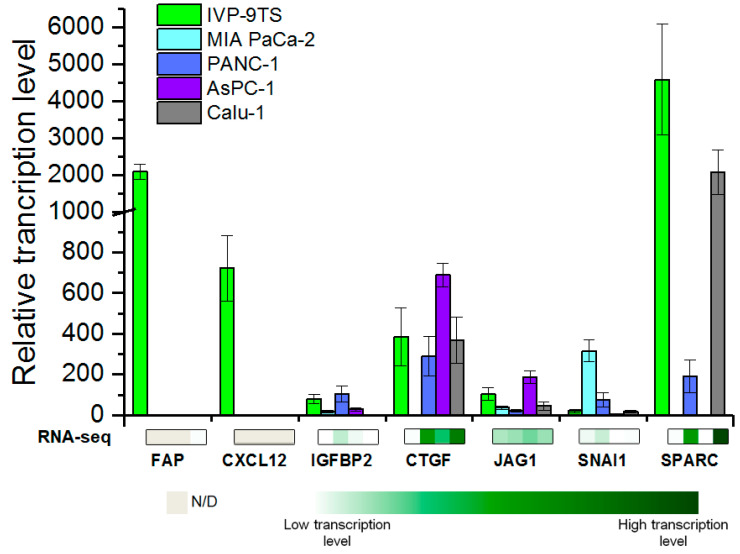
Determination of Transcription Level of selected genes in human cells. The Y-axis indicates the relative level of mRNA content in cells. The values obtained were normalized to the transcription levels of the 18S RNA. The measurements were performed in three independent experiments for each sample and are represented as mean ± s.e.m. The Y-axis serif designate scale break. Below the graph is a heat map of the expression values (in TPM, Transcripts Per Kilobase Million) of the genes in the studied cell lines according to the RNA-seq E-MTAB-2706 database. The names of genes are indicated below the heat map. N/D—no data available.

At the first stage of the work, we determined the transcription level of the *FAP*, *CXCL12. IGFBP2*, *CTGF*, *JAG1*, *SNAI1*, and *SPARC* genes in the primary culture of human fibroblasts IVP-9TS, in pancreatic cancer cell lines (MIA PaCa-2, PANC-1, AsPC-1) and lung cancer (Calu-1) cell line by quantitative real-time PCR (qPCR). The obtained transcription profiles of the *FAP*, *CXCL12*, *IGFBP2*, *CTGF*, *JAG1*, *SNAI1*, and *SPARC* genes in the studied cancer cell lines are comparable with the values of their expression according to the RNA-seq E-MTAB-2706 database (www.ebi.ac.uk, accessed on 30 June 2020) (Figure 1). This indicates the relevance of our data for cancer cell lines and increases the reliability of the data characterizing the primary culture of human fibroblasts IVP-9TS, as well as for genes whose expression data are not available in the database. 

The transcripts of the *FAP* and *CXCL12* genes were detected only in IVP-9TS primary culture of human fibroblasts, which is consistent with the literature data on their predominant expression in CAFs, but not in cancer cells [13,22]. For the *JAG1* and *IGFBP2* genes, a low transcription level was shown in all cell lines under the study. The transcription level of the *CTGF* gene was high and almost the same in all studied cell lines, except for MIA PaCa-2, in which it was absent. As expected, the transcription level of the *SNAI1* gene was low in most cancer cell lines, except for the MIA Paca-2. However, *SNAI1* expression was also almost absent in the IVP-9TS stroma fibroblasts. Transcription of the *SPARC* gene was higher in stroma fibroblasts (IVP-9TS) and lung cancer cells (Calu-1), compared to pancreatic cancer cell lines.

Thus, specific transcription in the primary culture of human fibroblasts IVP-9TS was shown only for the *CXCL12* and *FAP* genes.

### 2.3. The Choice of the Promoter Regions of the Studied Genes and Obtaining Reporter Constructs

**Figure 2:**
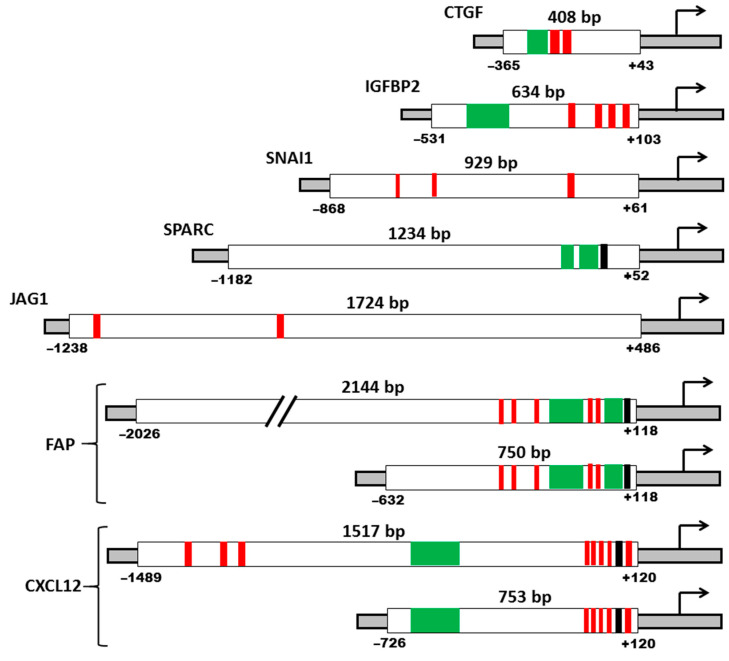
Schemes of promoter regions used in the work. Regions with several binding sites for transcription factors (TFs) are shown in green; deletion of such regions is critical for the activity of the promoter; sites of single binding TFs, important for the activity of the promoter, are marked in red; TATA-like regions are marked in black; the curved arrow indicates the translation initiation site.

Based on the literature analysis of data on the deletion analysis of promoters, we selected regions of promoters for obtaining reporter constructs (Figure 2). The promoter regions of most of the chosen genes are well studied (*FAP*, *CXCL12. IGFBP2*, *CTGF*, *SNAI1*, and *SPARC*). Whenever possible, the minimal region of the promoter that provide high activity was chosen. These regions contain the necessary and sufficient number of binding sites for transcription factors (TFs), able to provide the activity of the promoter. 

For the *FAP* and *CXCL12* genes, which were specifically transcribed in fibroblasts, two promoter fragments were chosen: the first, the minimal promoter, and the second, the promoter with distal promoter elements, in order to evaluate their contribution to the possible specific regulation of the promoter activity. 

The size of the *FAP* gene promoter is about 2000 bp. The core promoter controlling *FAP* expression is located within the 750 bp region, upstream the transcription initiation start. The most critical for transcription of the *FAP* gene is the proximal region of ~126 bp located upstream the transcription start site. This region provides the minimum level of transcription and contains the most important cis-regulatory elements (CREs) [30]. In this study, we used two regions of the FAP promoter. The first is 2144 bp full-length promoter that includes most of the regulatory elements. The second is 750 bp core promoter containing the most critical binding sites for transcription factors.

The length of the *CXCL12* gene promoter is about 1500 bp [31]. Deletion of the promoter region located between -601 and -512 bp relative to the transcription initiation site, leads to a strong inhibition of promoter activity, reducing it by ~95%. This region contains consensus sequences for the binding of transcription factors such as GCN4, XPF1, HF-X3/E12 [31]. A 753 bp promoter region was cloned, including the above-described key region for transcription. It was also decided to use a 1517 bp promoter fragment containing binding sites at the 5’-end for the c/EBPβ transcriptional activator, which plays an important role in the regulation of the *CXCL12* gene expression [32].

The *IGFBP2* gene promoter is less than 1000 bp length and is located in the CpG island. The region of about 640 bp upstream the first exon of the *IGFBP2* gene has promoter activity and does not contain TATA and CAAT sequences [33]. The transcription factor Egr-1 plays a significant role in the activation of the *IGFBP2* gene promoter; three binding sites for this factor were found in the promoter. In the proximal region of the promoter, there are binding sites for the Sp1 and NF-κB factors, and the E-box is located in distal part [33]. We have cloned a 634 bp fragment, which includes all of the above sites.

The *CTGF* gene promoter is about 800 bp; most of the identified binding sites for transcription factors are located in the proximal region extending for about 550 bp upstream the ATG site [34]. Based on the data that the distal region of the promoter does not significantly affect its work, we chose a region of 408 bp for cloning. It is the minimum region with promoter activity.

In contrast to other promoters used in this work, the *JAG1* gene promoter has hardly been studied and there are few data on its structure. Identification of the human *JAG1* gene promoter was based on bioinformatic methods [35]. As mentioned above, the Jagged1 functions as Notch signaling activator. Search for TCF/LEF-binding sites within the promoter region of Notch ligand genes identified them in the distal part of *JAG1* gene promoter, thus the total size of the promoter is considered to be about 1700 bp. Our analysis based on the UCSC Genome Browser database showed that this region contains the largest number of putative binding sites for TFs, and we used this region for cloning.

The *SNAI1* gene promoter contains an E-box essential for the regulation of expression by negative feedback [36], two HRE elements with binding sites for the factor HIF-1α [37], Smad elements of the TGFβ-signaling pathway [38], NFκB-sensitive element [39], sites AP-1, c-Jun, Egr1 [40]. A fragment of the promoter region of the *SNAI1* gene with a length of 929 bp containing most of the necessary regulatory elements was cloned [36].

Transcription of the *SPARC* gene requires CREs, the most important of which are purine-rich sequences—GGA-boxes, connected to each other by a 10-nucleotide spacer. The first GGA-box provides maximum promoter activity, while the pyrimidine-rich spacer region is responsible for negative regulation [41]. Based on the previously obtained literature data on the in vitro activity of several fragments of the human *SPARC* gene promoter [41,42], we chose a 1234 bp promoter region for cloning.

**Figure 3:**
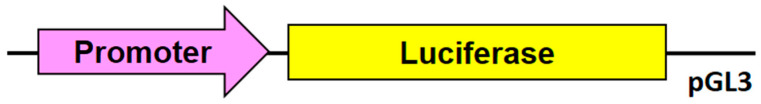
Scheme of the reporter constructs (based on pGL3). Promoter—FAP, CXCL12, IGFBP2, CTGF, JAG1, SNAI1, or SPARC, which are the promoters of the respective genes. Luciferase: firefly *luciferase* gene.

For promoter activity study, we prepared reporter constructs containing the *luciferase* gene under the control of various promoters (Figure 3). The above chosen promoter regions of the *FAP*, *CXCL12*, *IGFBP2*, *CTGF*, *JAG1*, *IGFBP2*, *SNAI1*, and *SPARC* genes were used. The constructs differed only in promoters. A detailed cloning scheme for each construct is presented in Section 4.2. Construction of expression vectors. Further in the text, the names of the promoters of the corresponding genes are indicated in capital letters.

### 2.4. Evaluation of Promoter Activity

To evaluate the activity of the studied promoters—FAP 0.75, FAP 2.2, CXCL12 0.7, CXCL12 1.5, IGFBP2, CTGF, JAG1, SNAI1, SPARC (see Section 4.1. Promoters used, for detailed description), transient transfections with the obtained reporter constructs for the primary culture of human fibroblasts IVP-9TS, cell lines of pancreatic (MIA PaCa-2, PANC-1, AsPC-1) and lung cancer (Calu-1) were performed. 

Plasmid pGL3-PV containing the standard weak nonspecific SV40 promoter and pGL3-Basic vector, a promoterless plasmid, were used as control constructs in the experiment. To evaluate the relative strength of the promoters, a reporter construct containing a strong viral nonspecific cytomegalovirus immediate-early promoter (CMV) known as the “gold standard” of a highly active promoter was used as a control. A construct containing the well-studied cancer-specific promoter of the human *Survivin* (*BIRC5*) gene (designated as SURV) [43,44], was taken as a control promoter providing expression in cancer cells.

**Figure 4:**
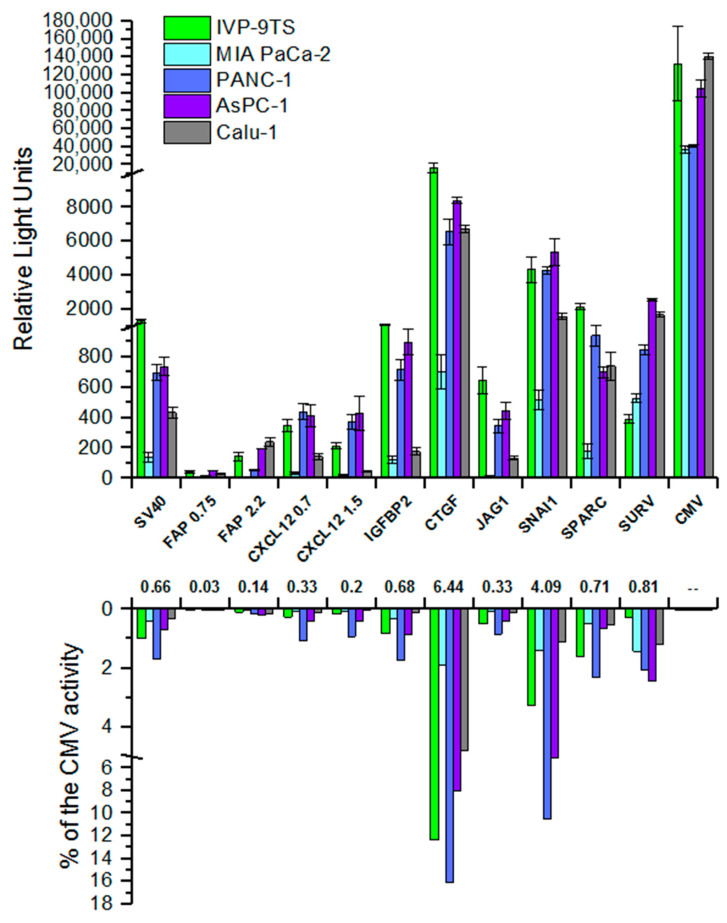
Relative promoter activities of the studied promoters in different human cell lines. The upper graph represents relative promoter activities (Y-axis) as ratios of the luciferase activity expressed by plasmids containing promoters under study to the R. reniformis luciferase activity. Luciferase activity expressed by promoterless pGL3-basic plasmid was subtracted. Mean values (±s.e.m.) of relative luciferase activity were calculated from three independent experiments. The Y-axis serifs designate scale breaks. The names of promoters are indicated below the top X-axis and correspond to both graphs. The numbers under the names of promoters indicate the median activity of promoters in cell lines relative to the activity of the CMV promoter. The lower graph represent the activity of the promoters under the study (Y-axis, expressed in %) relative to the activity of the CMV promoter in each cell line. Graph for the CMV activity (100%) is omitted.

Obtained results for transient transfection of various cells showed that the studied promoters are active in all studied cell lines (Figure 4, upper graph). When normalizing the activities of the studied promoters to the activity of the universal CMV promoter, it was shown that the activity for most promoters was several percent of its activity in the same cells (Figure 4, lower graph). The lowest activity of most promoters was observed in the MIA PaCa-2 cell line. 

As expected, the cancer-specific SURV promoter demonstrated a rather high activity in cancer cells as compared to fibroblasts.

The IGFBP2, CTGF, JAG1, and SPARC promoters are most active in the primary culture of human fibroblasts IVP-9TS, while the IGFBP2 and JAG1 promoters virtually are not active in the epithelial cells of the lung cancer Calu-1. However, we observe the same increased activity in the IVP-9TS for the weak nonspecific SV40 promoter. Moreover, the activity of the strong universal CMV promoter in the primary culture of human fibroblasts is, on average, 2–3 times higher than in pancreatic cancer cell lines, but comparable to the activity in lung cancer cells (Calu-1). Unexpected results were obtained using constructs containing the CXCL12 and FAP promoters, which showed high specific transcription in the primary culture of human fibroblasts. It turned out that the activity of these promoters is unspecific and is the weakest among all the studied promoters.

We used the human umbilical vein endothelial cells (HUVEC) as a control non-cancer cell line to determine the selectivity of the “tumor on” phenotype of the promoter [45]. All promoters show no activity in the HUVEC cell line, except for the CMV, which was expected, and a lower promoter activity compared to the CMV activity for the CTGF. We suppose that this is due to the very high level of endogenous transcription of the *CTGF* gene in these cells (data not shown).

Although the luciferase expression by reporter constructs containing regions of various promoters showed different activities in the panel of cell lines, the promoters could be divided into three groups, according to the level of their activity:

- With low activity (up to 1000 relative light units (RLU), weaker than the SV40 promoter)—FAP, CXCL12, JAG1.
- With medium activity (about 1000 RLU, comparable to SV40)—IGFBP2, SPARC, SURV.
- With relatively high activity (more than 4000 RLU, stronger than SV40)—SNAI1, CTGF.

We were searching for the promoters that are active selectively in CAFs. Our results indicate that there are no such selectivity for promoters under the study. Most likely, transcriptional targeting suffers from the problems similar for molecular targeting of surface markers—the lack of selective CREs. At least if the minimal region of the promoter that provide its high activity was chosen as CRE. 

Notwithstanding, we noticed that: (i) promoters could be grouped by strength, which could be represented as the median activity of promoter; (ii) universal promoters in CAFs could work better than in cancer cells. We suggest that the ratio of the median activity of promoters relative to each other could be relatively stable for conservative promoters. We chose one promoter from each of three groups (CTGF, IGFBP2, FAP 2.2) for whose the median activity differs by an order of magnitude to examine our hypothesis. 

Since we are interested to use promoters for gene therapy, we investigate our suggestion in the mouse model. 

### 2.5. Intercomparison of In Vitro and In Vivo Promoter Activities

#### 2.5.1. Evaluation of the Degree of Identity for Human and Mouse Homologous Sequences

Currently, little is known about how the activity of promoters in artificial constructs is related in vitro and in vivo. We compared the changes in the median promoter activity for different promoters during the transition from one system to another, as well as compared their activities relative to each other and the strong universal CMV promoter. To ensure that our results can be used in mouse models or translational research, we have determined the luciferase activity levels from previously used constructs containing human gene promoters in both mouse cell lines and in vivo mouse model.

For further study, we should choose the promoters with the highest degree of identity between human and mouse in order to avoid the contribution of species-specific transcription factors. To determine the degree of identity of the studied mouse and human promoters of homologous genes, a bioinformatic analysis of the structure of the promoters was performed (Table 1).

Based on previous data, we hypothesized that the ratio of the median activity of promoters relative to each other could be relatively stable for conservative promoters. The CTGF, IGFPB2, and FAP 2.2 promoters were chosen from three groups that differed by orders of magnitude in median activity in human cell lines. The constructs with the strong universal CMV promoter and the cancer-specific SURV promoter were taken as control promoters. We have shown above, that universal promoters can provide a higher level of gene expression in fibroblasts. To examine this, along with the universal nonspecific viral CMV promoter, we took the previously studied universal mammalian promoter PCNA [46].

#### 2.5.2. Evaluation of the Promoter Activities in Mouse Cell Lines

**Figure 5:**
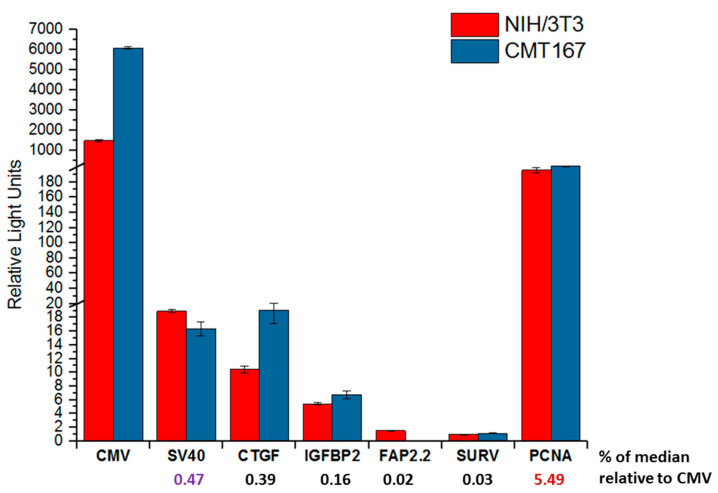
Relative promoter activities of the studied promoters in mouse cell lines. The graph represents relative promoter activities (Y-axis) as ratios of the luciferase activity expressed by plasmids containing promoters under study to the R. reniformis luciferase activity. Luciferase activity expressed by promoterless pGL3-basic plasmid was subtracted. Mean values (± s.e.m.) of relative luciferase activity were calculated from at least three independent experiments. The Y-axis serifs designate scale breaks. The names of promoters are indicated below the X-axis. The numbers under the names of promoters indicate the median activity of promoters in mouse cell lines relative to the activity of the CMV promoter. Median promoter activities higher than SV40 (purple) are shown in red.

To examine the possible contribution of species-specific TFs, we determined the activity of the studied promoters in murine cell lines. For this, the mouse lung cancer cell line CMT 167 (the cells could be used to obtain mice with inoculated syngeneic tumor) and the mouse fibroblasts NIH/3T3 were employed. Transfection and data analysis was performed as in the case of human cell lines. The values of the promoter activities are shown in the Figure 5.

Analysis of the results obtained showed that all promoters with respect to CMV exhibit low activity. This was expected due to the calculated values of median activity relative to CMV promoter for human cell lines. The activity of all studied promoters was at least an order of magnitude lower than in human cell lines. The activity of promoters in cancer cells and fibroblasts differed little, and can be represented as the median activity. The median activity of the promoters decreases in the row CTGF, IGFBP2, and FAP 2.2, similar to what was shown previously for human cell lines. However, the level of changes in the promoter activity was other; the changes were in 2–3 times, but not by an order of magnitude, as in the case of human cells. We suggest that this could be because the degree of identity of human and mouse promoters is about 65–80%, which reduces the efficiency of human promoters in mouse model.

The SURV activity was as low as for the FAP 2.2 promoter. Such a low activity of the human SURV promoter in mouse cell lines may be associated with a significant difference in the structure of the human and mouse *Survivin* (*BIRC5)* gene promoters (see Table 1).

We demonstrate that promoter activity of the PCNA promoter is high and the same in cancer cells and fibroblasts.

#### 2.5.3. In Vivo Analysis

To understand whether our results can be used for translational studies, we carried out a study of the activity of promoters in a grafted tumor model. For in vivo experiments, a transplantable tumor model of mouse lung carcinoma CMT 167 was chosen as subcutaneous model for C57BL mice. We used intratumoral administration of constructions, by which all types of tumor cells could be transfected. Since earlier we did not find a fundamental difference in the activity of promoters in various cells, this model was chosen as the most convenient for the study. We have previously examined the efficiency of transfection for different mouse models (to be published elsewhere). It was shown that in the CMT 167 model the transfection level is the highest, which is important, since other promoters exhibit very low activity, and in vivo their activity could be lower than the sensitivity level of the method.

**Figure 6:**
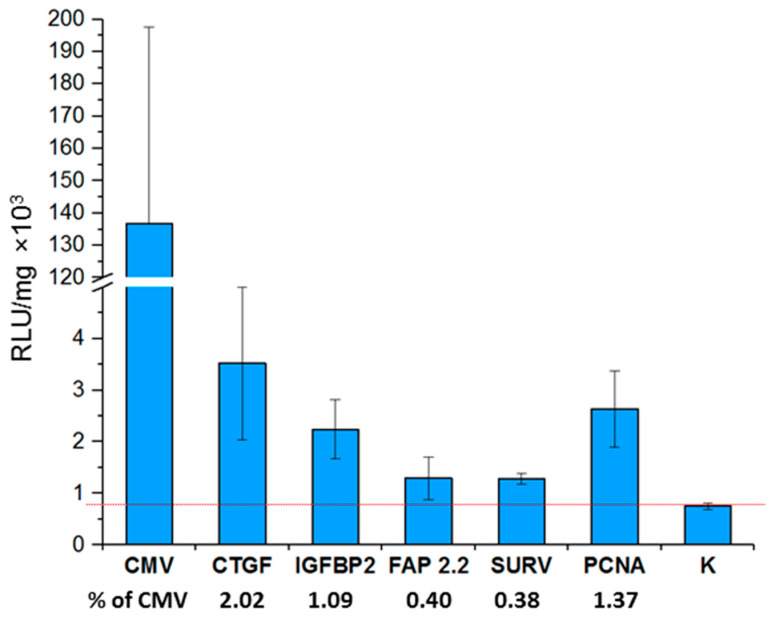
Relative promoter activities of the studied promoters in mouse CMT 167 tumors. The graph represents relative promoter activities (Y-axis) as ratios of the luciferase activity expressed by plasmids containing promoters under the study normalized to milligram of protein. The Y-axis serifs designate scale breaks. The numbers under the names of promoters indicate the activity of promoters in tumors relative to the activity of the CMV promoter. The measurements were performed in 3–4 technical replicates for each sample and are represented as mean ± s.e.m. RLU/mg—relative light units per milligram of protein.

In total, seven groups of mice were formed (3 mice per group)—one group for each construct and a control group of animals. C57BL mice were subcutaneously inoculated with 10^6^ CMT 167 lung cancer cells at both flanks. When the tumors reached the size of 50–120 mm^3^, the corresponding constructs were injected intratumorally. A polyplex previously examined by us for gene therapy purposes was used as a non-viral vector for the delivery of constructs into cells [7]. After 48 h, mice were sacrificed, tumors collected and used to determine the level of luciferase activity; the relative light units (RLU) values were normalized to the amount of total protein (Figure 6).

The activity of promoters decreases in the series of CTGF, IGFBP2, and FAP 2.2/SURV. The activity pattern of these promoters completely repeat the activity patterns observed by us during in vitro transfection of mouse cell lines. The ratio of the activity of these promoters is conserved both among themselves and relative to the activity of the universal CMV promoter.

An exception is the activity of the PCNA promoter, which in the case of in vitro showed higher values of activity than in vivo. We hypothesize that this may be either due to contact inhibition of cell growth in the tumor (PCNA is a well-known proliferation marker), or due to tumor hypoxia, it is assumed that the activity of the PCNA promoter is inhibited during hypoxia [47,48]. On the other hand, the expression of the *CTGF* gene is activated by hypoxia [49]. Thus, in addition to the median activity, the features of the promoter itself may contribute when comparing the activity of the promoters in vivo and in vitro. 

## 3. Discussion

The rapid development of gene therapy drugs with non-viral vectors is restrained by such factors as ineffective transfection of target cells and a low level of transgene expression. Intratumoral administration of such drugs can lead to transfection of all types of tumor cells—the cancer cells, immune cells of the tumor, and cancer-associated fibroblasts. The latter are of interest as a long-term source for the synthesis of therapeutic genes. The possibility of transcriptional targeting of transgene expression in CAFs is a poorly studied area. Little is known about which tumor cells are primarily subjected to gene therapy transformation and, as far as possible, the implementation of transcriptional targeting. The analysis of transfected populations of cells in a tumor in vivo is quite complex and elusive. Therefore, as a model for in vitro study, the primary culture of human fibroblasts and cancer cell lines of different lineage, were chosen.

The aim of our study was to reveal the possibility to use promoters of the genes with increased expression in the TME for transcriptional targeting of transgene expression in CAFs. During the study of gene transcription in a panel of human cancer cell lines and primary culture of human fibroblasts, we identified the *CXCL12* and *FAP* genes that showed high and specific transcription in fibroblasts. Transcription of the *IGFBP2*, *CTGF*, *JAG1*, and *SPARC* genes was less specific, but in most cases it was increased in fibroblasts.

It is well known that the population of CAFs is highly heterogeneous, which is due to both the different origin and stage of differentiation of cells, as well as because of functional heterogeneity of CAFs subpopulations in the tumor stroma [14]. All of these factors results in the differences in gene expression patterns. For example, the expression level of highly specific CAFs markers such as αSMA and FSP1 differs in various CAFs subpopulations in the case of oral squamous cell carcinoma [50] and rectal cancer [51]. Therefore, the lack of specificity of transcription of other studied genes only indicates that the primary fibroblasts IVP-9TS used in the experiment are characterized by such an expression pattern. Due to the difficulty of obtaining and the limited number of cell divisions, we were limited to only one line of fibroblasts—the primary culture of human fibroblasts IVP-9TS. In this primary culture, a unique transcription of the *CXCL12* and *FAP* genes was observed; by pattern of gene expression, they corresponded to fibroblasts, and the SURV promoter of the cancer-specific gene *Survivin (BIRC5)* worked in it worse than in cancer cells. Thus, we conclude that IVP-9TS was a relevant and sufficient model of CAFs.

We hypothesized that promoters of genes with increased expression in stroma may maintain selective promoter activity in CAFs. We used artificial constructs, where such promoters were used to control the expression of the reporter *luciferase* gene. For example, previously published works indicate the activity of the FAP promoter as part of an artificial construct in FAP-positive cell lines, where the required TFs are present [30,52]. To test our hypothesis, a series of transient transfections of cell lines was performed.

We have demonstrated that almost all of the studied promoters (except for CTGF and SNAI1) exhibit extremely low levels of promoter activities. We found selective promoter activity in the primary culture of human fibroblasts for none of the studied promoters. Even in the case of the promoters of the *CXCL12*, *FAP*, and *SPARC* genes, which showed high transcription levels in IVP-9TS as compared to cancer cell lines. For the promoters of the *CXCL12* and *FAP* genes, the transcripts of whose were detected specifically in IVP-9TS fibroblasts, an extremely low and nonselective promoter activity was shown both in IVP-9TS and in cancer cell lines. This was unexpected, especially in the case of the *FAP* gene promoter. Despite the fact that some works indicate the importance of the distal CREs of the *FAP* gene promoter for its tissue-specific activity [30,52], we did not find any difference in the selectivity of the promoter activity for the two selected regions of the *FAP* gene promoter.

It is worth noting that the CTGF promoter activity was highest among all studied promoters. The observed activity of the CTGF promoter in the endothelial cell line HUVEC may suggest that the promoter could belong to endothelial cell-specific promoter class [53]. These type of promoters of specific genes that are upregulated in proliferating endothelial cell are attractive for targeting transgenes to the tumor vasculature. CTGF is barely expressed in normal adult tissue, but is strongly upregulated in fibrotic tissue and is also increased during development, in wound healing, or in certain types of cancer. Accordingly, gene expression of *CTGF* is tightly regulated [54]. The high activity of this promoter in endothelial, cancer cells, and CAFs, suggests that it may be selective specifically for tumor and tumor vasculature, although we do not know about its selectivity for normal non-vasculature tissues. However, this requires further studies.

We did not find any correlations between the high level of endogenous gene transcription and the activity of the corresponding promoters in the reporter constructs. This generally confirms the previously published work, which showed that the high activity of the *Survivin* gene promoter in the reporter constructs do not correlate with the high level of the native Survivin protein in corresponding cell lines [45]. 

When analyzing the activities of promoters on human cells, we had divided the studied promoters into several groups according to the level of activity. In the absence of a pronounced selectivity of the promoters, we suggested that each of the studied promoters could be characterized by its own median level of activity in all used cell lines (median activity). We believe such median activity could be a characteristic of each promoter, which is possibly conserved in various types of cells, and depends little on their lineage. The median activity of a promoter can serve as its characteristic, which depends on the structure of the promoter, the composition of binding sites for TFs. Thus, the most important for the median activity of the promoter is the possibility of using a wide range of transcription factors. 

Really, the universal viral promoters CMV and SV40 showed a higher level of activity in fibroblasts as compared to cancer cells. This fact, as well as the shown low activity of most promoters, suggests that the universal promoters may be suitable for providing expression in CAFs, although this may sacrifice the selectivity for promoter activity. Other drawbacks of many universal promoters is silencing, even when administered as part of artificial constructions by non-viral vectors [55,56]. Thus, in addition to promoters that are active in CAFs and have a certain set of TFs characteristic of this cell type, we took the universal mammalian PCNA promoter. Its TFs pattern is associated with the cell replication apparatus, a more universal feature (cell division) than selective expression in a particular cell type, and which is less subjected to silencing than the CMV [46].

We have shown that the concept of median promoter activity is retained in vivo, at least partially and upon short-time transgene expression. This is a fundamental point. We speculate that the median promoter activity compared to CMV activity (or other known promoter) may offer insights for the promoter activity in vivo. The degree of identity and features of the regulation of the promoter should also be taken into account. Thus, it is difficult to expect high promoter activity for weak promoters such as FAP, but it is possible for strong promoters like CTGF and PCNA. In addition, the results obtained suggest that additional CREs should be used to provide selective promoter activity. 

The results obtained showed that:

1. No correlation was found between the high level of native gene transcription in cells and the activity of the corresponding promoters in reporter constructs during transfection of the same cells. The promoters of genes that showed the highest transcriptional specificity in fibroblasts (*CXCL12* and *FAP*) have the lowest and nonspecific activity when used in artificial reporter constructs.
2. The promoters used in this work could be divided into three different groups by their strength (the median activity characterizing each promoter). The CTGF promoter was the strongest promoter among the studied promoters. The ratios of the promoter activity for different promoters in reporter constructs can persist both when used in vitro and in vivo, including for different species. We have shown this in our study under conditions of short-term expression, at least for conservative promoters (a relatively high degree of identity of human vs mice promoters).
3. To ensure transcriptional targeting it is not enough to use only promoters. Other CREs (enhancers, specific regulatory elements, etc.) should also be used.
4. Intratumoral administration of therapeutic constructs can lead to transfection of all types of tumor cells. Based on our in vitro results, we suggest that the level of transgene expression in fibroblasts compared to cancer cells can be higher when using universal promoters (CMV, SV40, PCNA).

Apparently, the search for promoters providing transcriptional targeting in CAFs may be in vain when it is required to provide selective transgene expression in them. In general, we have to admit that transcriptional targeting of CAFs has limitations similar to the targeting of surface markers. It is possible to achieve a high level of transgene expression in fibroblasts, but sacrifice selectivity. As in the previous work [46], we assume that the most optimal is the use of universal promoters. The concept of conserved median promoter activity demonstrated in this work, suggests that, for gene therapy purposes, it is possible to follow the path of creating chimeric promoters in which a strong universal promoter will be combined with CREs, which could increase the activity or selectivity of the promoter. For example, in our work, the pan-cancer TS269 promoter was obtained by fusion of strong cancer promoters of the *TERT* and *BIRC5* genes [44]. Possibly, the CREs of specific promoters can be used to increase the activity and selectivity of strong promoters. However, this requires further research.

## 4. Materials and Methods

### 4.1. Promoters Used

The FAP used was the promoter of the human *fibroblast activation protein* (*FAP*) gene. For cloning and further study, we used two fragments of the FAP promoter, FAP 0.75 (750 bp) and FAP 2.2 (2144 bp) with coordinates −2026/+118 and −632/+118 relative to the transcription start site (TSS), respectively.

The CXCL12 used was the promoter of the human *C-X-C motif chemokine ligand 12* (*CXCL12*) gene. For cloning and further study, we used two fragments of the CXCL12 promoter, CXCL12 0.7 (753 bp) and CXCL12 1.5 (1517 bp) with coordinates −1397/+120 and −633/+120 relative to the TSS, respectively.

The IGFBP2 used was the promoter of the human *insulin-like growth factor binding protein 2* (*IGFBP2*) gene. For cloning and further study, we used a fragment of the IGFBP2 (634 bp) promoter with coordinates −531/+103 relative to the TSS. 

The CTGF used was the promoter of the human *connective tissue growth factor* (*CTGF*) gene. For cloning and further study, we used a fragment of the CTGF (408 bp) promoter with coordinates −365/+43 relative to the TSS.

The JAG1 used was the promoter of the human *jagged canonical Notch ligand 1* (*JAG1*) gene. For cloning and further study, we used a fragment of the JAG1 (1724 bp) promoter with coordinates −1238/+486 relative to the TSS.

The SNAI1 used was the promoter of the human *snail family transcriptional repressor 1* (*SNAI1*) gene. For cloning and further study, we used a fragment of the SNAI1 (929 bp) promoter with coordinates −868/+61 relative to the TSS.

The SPARC used was the promoter of the human *secreted protein acidic and cysteine rich* (*SPARC*) gene. For cloning and further study, we used a fragment of the SPARC (1234 bp) promoter with coordinates −1182/+52 relative to the TSS.

The SURV used was the promoter of the human *baculoviral IAP repeat containing 5* (*BIRC5*) gene. For further study, we used a fragment of the SURV (1498 bp) promoter, that was earlier studied in our laboratory [43,44] and is considered a universal cancer promoter.

The PCNA used was the promoter of the human *proliferating cell nuclear antigen* (*PCNA*) gene. In our study, we used a PCNA short promoter (389 bp, coordinates −241/+148 with respect to the TSS) that was earlier studied in our laboratory [46,57] and is considered a strong universal promoter for mammalian cells.

The CMV used was CMV Pr/Enh promoter containing an AseI/BglII fragment of the promoter of early cytomegalovirus genes from plasmid pEGFP-N1 (Clontech Laboratories (Takara Bio), Shiga, Japan) [58].

### 4.2. Obtaining of Reporter Constructs

To obtain reporter constructs, chosen regions of promoters were amplified from the human genomic DNA using specific primers (Table 2) and Q5 High-Fidelity DNA Polymerase (New England Biolabs, Ipswich, MA, USA) or Tersus Plus PCR kit (Evrogen, Moscow, Russia). 

To obtain FAP 2.2-luc-pGL3 plasmid the promoter FAP 2.2 was amplified by PCR using the primers FAP-for-2145/FAP-NcoI-Rev and subcloned to the pAL-2T vector (Evrogen) by TA-ligation. Fragment containing FAP 2.2 was excised from the pAL-2T vector by SacII (blunted)/NcoI restriction endonucleases and cloned into a pGL3-Basic Vector digested with HindIII (blunted)/NcoI restriction endonucleases by sticky-blunt ligation. To obtain FAP 0.75-luc-pGL3 plasmid the promoter FAP 0.75 was amplified from FAP 2.2-luc-pGL3 plasmid by PCR using the primers FAP-HindIII-for/FAP-NcoI-Rev and cloned into a pGL3-Basic vector digested with HindIII/NcoI restriction endonucleases by sticky-end ligation.

To obtain CXCL12 1.5-luc-pGL3 plasmid, the promoter CXCL12 1.5 was amplified by PCR using the primers CXCL12-HindIII-for/CXCL12-NcoI-Rev and cleaved by HindIII/NcoI restriction endonucleases. The excised fragment was cloned into a pGL3-Basic vector digested with HindIII/NcoI. To obtain CXCL12 0.7-luc-pGL3 plasmid, the promoter CXCL12 0.7 was amplified from CXCL12 1.5-luc-pGL3 plasmid by PCR using the primers SDF1-F630/CXCL12-NcoI-Rev. The amplification product was cleaved by NcoI restriction endonuclease and cloned into a pGL3-Basic vector digested with SmaI/NcoI restriction endonucleases.

To obtain CTGF-luc-pGL3 plasmid the promoter CTGF was amplified by PCR using the primers CTGF-HindIII-for/CTGF-NcoI-Rev and cleaved by HindIII/NcoI restriction endonucleases. The excised fragment was cloned into a pGL3-Basic vector digested with HindIII/NcoI.

To obtain JAG1-luc-pGL3 plasmid the promoter JAG1 was amplified by PCR using the primers JAG1-HindIII-for/JAG1-NcoI-Rev and cleaved by HindIII/NcoI restriction endonucleases. The excised fragment was cloned into a pGL3-Basic vector digested with HindIII/NcoI.

The fragment of the SNAI1 promoter was taken from modified plasmid pGL3 kindly provided by the laboratory of Dr. Antonio Garsia de Herreros (Spain). To obtain SNAI1-luc-pGL3 plasmid the fragment containing SNAI1 promoter was excised by KpnI/HindIII restriction endonucleases and cloned into a pGL3-Basic vector digested with KpnI/HindIII. 

To obtain SPARC-luc-pGL3 plasmid the SPARC promoter was amplified by PCR using the primers SPARC-F2-For/SPARC-R72-NcoI-Rev, subcloned to the pAL-2T vector (Evrogen) by TA-ligation. Fragment containing SPARC promoter was excised by NcoI restriction endonuclease and cloned into a pGL3-Basic vector digested with NcoI.

The SURV-luc-pGL3 plasmid with the SURV promoter was obtained earlier [43,44]. The PCNA-luc-pGL3 plasmid with the PCNA promoter was obtained earlier [57]. The IGFBP2-luc-pGL3 plasmid with the IGFBP2 promoter was obtained earlier [46].

All restriction endonucleases were purchased from Thermo Fisher Scientific (Waltham, MA, USA). All plasmids were isolated by Endofree Plasmid Midi or Giga Kit (Qiagen, Venlo, The Netherlands) according to the manufacturer’s protocol. The structure of all plasmids obtained was confirmed by restriction analysis and sequencing. All sequences of the promoters used could be found in Supplementary S1.

### 4.3. Cell Lines

Cancer cell lines derived from human pancreatic cancer—MIA PaCa-2 (ATCC^®^ CRL-1420), PANC-1 (ATCC^®^ CRL-1469), AsPC-1 (ATCC^®^ CRL-1682); human umbilical vein endothelial cells—HUVEC (ATCC^®^ CRL-1730); non-small-cell lung cancer cell line Calu-1 (ATCC^®^ HTB-54™) and mouse embryonic fibroblast cell lines NIH/3T3 (ATCC^®^ CRL-1658™) were obtained from the American Type Culture Collection (ATCC, Manassas, VA, USA). Mouse lung carcinoma CMT 167 (clone of CMT 64, ECACC 10032302) was obtained from European Collection of Cell Cultures (ECACC, Salisbury, UK). 

A primary cell culture of human fibroblasts, IVP-9TS, was provided by the Vishnevsky Institute of Surgery (Moscow, Russia) [17].

MIA PaCa-2, PANC-1, AsPC-1, NIH/3T3, Calu-1, CMT 167 cell lines, and IVP-9TS fibroblasts were cultured in DMEM/F12 (1:1) medium supplemented with 100 units/mL penicillin, 100 µg/mL streptomycin, and 0.25 µg/mL amphotericin and 10% fetal bovine serum. The media and supplements were purchased from Gibco (Thermo Fisher Scientific). HUVEC cell line were cultured by BEGM Bronchial Epithelial Cell Growth Medium BulletKit (Lonza, Basel, Switzerland) supplemented with 2% fetal bovine serum. Cells were maintained in a humidified atmosphere at 5% CO_2_ and 37 °C.

### 4.4. Transfection of Cells

Cells were transfected in 24-well plates using Lipofectamine 2000 (Invitrogen, Thermo Fisher Scientific, Waltham, MA, USA) according to the manufacturer’s recommendations. Transfection was done with 1 mkg (per well) mixture of a reporter plasmid carrying the firefly *luciferase* gene and an internal control plasmid pRL-TK (Promega, WI, USA) in the weight ratio of 9:1. In 48 h after transfection, the activity of firefly and Renilla reniformis luciferases was measured in cell extracts using a Dual-Luciferase Reporter Assay System (Promega) and a GENios Pro luminometer (Tecan, Männedorf, Switzerland). In parallel experiments, cells were transfected with a promoterless pGL3-Basic plasmid or a PV-pGL3 plasmid, containing only the SV40 promoter. For each construct under the study, at least three independent transfections were performed. The luciferase activity was normalized to that of BV-pGL3 Basic vector activity. Calculations were made using the MS Excel 2013 program.

### 4.5. RNA Isolation

To isolate total RNA cells were trypsinised and washed twice with phosphate-buffered saline (PBS). Then, the cell pellet was collected and stored at −70 °C prior to isolation of RNA.

Total RNA was isolated from 1 million cells using an RNeasy Mini Kit (Qiagen) followed by treatment with DNAse RQ1 (Promega) according to the manufacturer’s protocol. The quality of RNA was analyzed by electrophoresis in a 1% agarose gel containing ethidium bromide. The amount of RNA was determined with a NanoDrop 2000 spectrophotometer (Thermo Fisher Scientific) at the absorption wavelength of 260 nm.

### 4.6. Transcription Analysis by qPCR

The transcription level of the genes under the study in different cell lines and IVP-9TS primary culture was evaluated by qPCR using a qPCRmix-HS SYBR reaction mixture (Evrogen). The first cDNA strands were synthesized using hexanucleotide primers and Mint reverse transcriptase (Evrogen) according to the manufacturer’s protocol. For this purpose, total RNA was isolated from the cell pellets of the corresponding cell lines. A gene specific primer pair (see Table 2), was used to determine the transcription level of the corresponding genes. Data were normalized relative to the transcription level of the 18S RNA. Statistical processing of the data was performed using MS Excel 2013, LinRegPCR, and LC480Conversion.

### 4.7. In Vivo Assay

The C57BL mice (6–8 weeks) were obtained from the Pushchino Animal Breeding Facility (branch of the Shemyakin-Ovchinnikov Institute of Bioorganic Chemistry, Russian Academy of Sciences). The studies using mice were reviewed and approved by the Shemyakin-Ovchinnikov Institute of Bioorganic Chemistry Institutional Animal Care and Use Committee (IACUC, protocol No. 244, 17 December 2019). All animal manipulations were performed according to the recommendations of the European Convention for the Protection of Vertebrate Animals Used for Experimental and Other Scientific Purposes, Council of Europe (ETS 123). For tumor generation, 100 μL of PBS solution containing 10^6^ of the CMT 167 cells were injected subcutaneously into both flanks of the lower part of the mouse body. This was done in accordance with the 3R principles to reduce the number of animals in the experiment. After 10 days, when the tumors reached the size of 50–120 mm^3^, a copolymer-DNA complexes containing corresponding plasmids were injected intratumorally in dose of 0.08 mkg DNA/mm^3^. A polyethylenimine (PEI)-polyethylene glycol (PEG)-TAT peptide copolymer (PPT) was used to obtain copolymer-DNA complexes, as described earlier [7]. After 48 h after intratumoral injection mice were euthanized and tumors were collected.

### 4.8. Preparation of Tumor Lysates

Each tumor was placed in Lysing Matrix A tube (MP Biomedicals, Irvine, CA, USA) and 0.5 mL of PLB 1X buffer (Promega) was added. The tumor was grinded and lysed by FastPrep-24 machine (MP Biomedicals). The aliquots of the lysates were used to determine the luciferase activity and amount of protein by the Bradford method.

### 4.9. Luciferase and Bradford Protein Assays

To measure the amount of protein, the samples were mixed with a dye reagent in the ratio of 4:1, and incubated at room temperature for 10 min with constant stirring. The absorbance was measured with the Benchmark™ Plus Microplate Reader (Bio-Rad, Hercules, CA, USA) at 595 nm. The activity of firefly luciferase in cell extracts was measured using a Dual-Luciferase Reporter Assay System (Promega) and GENios Pro (Tecan, Switzerland) luminometer. The luciferase activity was normalized to that of 1 mg of protein. Calculations were made using MS Excel 2013 program.

### 4.10. Promoter and Gene Alignment

To evaluate the degree of identity for human and mouse homologous genes and their promoters corresponding sequences were extracted from UCSC Genome Browser database. The following database assembly was used—Human Dec. 2013 (GRCh38/hg38) and Mouse Jun. 2020 (GRCm39/mm39).

Alignment of the human and mouse promoter sequences was performed for region upstream the untranslated regions (UTR) using the AlingX Vector NTI program. First, the 2.5 kb regions upstream the UTR were aligned and the region of maximum identity was found. After that, the regions corresponding to the fragment of the used promoter were aligned. The coordinates were determined using a fragment of the promoter of the corresponding human gene, then the same coordinates were used for the mouse promoter, alignment was performed, and then the zone of maximum overlap was found—“max identity”. These are the best matching overlapping promoter regions.

To align the gene sequences, the CDS (coding sequence) of the corresponding genes were taken. If there were several transcript variants, the closest ones were selected. Predicted Protein sequence from UCSC Genome Browser was taken to align proteins. Alignment was performed by NCBI BLASTP tool (Identities).

## Figures and Tables

**Table 1:** The degree of identity for human and mouse homologous sequences.

Gene	Human vs. Mouse Identity, %			
	Promoter Region Max Identity		Gene	
	Overlap *	2.5 kb ^2^*	Nucleotide Sequence	Protein
*FAP*	70.2 (FAP 2.2)	66.5	89.4	90
	77.4 (FAP 0.75)			
*CXCL12*	51.2 (CXCL12 1.5)	<50	90.4	93
	55.4 (CXCL12 0.7)			
*IGFBP2*	65.3	51.3	81.7	83
*CTGF*	78.1	62.7	88	91
*JAG1*	79.9	62.9	90.2	97
*SNAI1*	50.7	<50	85	88
*SPARC*	52.1	<50	88.9	93
*BIRC5*	<50	<50	80.9	84

* shows the maximum degree of identity for the overlapping human vs mouse sequences of the promoter used. ^2^* shows the maximum degree of identity for the 2.5 kb upstream region relative to 5′-UTR. The promoters with the highest degree of identity are marked in green.

**Table 2:** Primers used in the experiments.

Primers Used in qPCR		
Gene	Primer	Primer sequence
*FAP*	FAP-for-S_E15	5′- CAGCAAGTTTCAGCGACTAC -3′
	FAP-rev-S_E19	5′- CAGCAAATACAGACCTTACAC -3′
*CXCL12*	CXCL12-forE2	5′- TCAGCCTGAGCTACAGATGC -3′
	CXCL12-revE3	5′- AGCTTCGGGTCAATGCACAC -3′
*IGFBP2*	IGFBP2-forE4	5′- AGATGTCTCTGAACGGGCAG -3′
	IGFBP2-revE4.1	5′- AAGAGATGACACTCGGGGTC -3′
*CTGF*	CTGF-ForE2.1*	5′- CGCACAAGGGCCTCTTCTG -3′
	CTGF-RevE3.1	5′- GAACGTCCATGCTGCACAG -3′
*JAG1*	JAG1-forE2	5′- AGTGTGCCTCAAGGAGTATC -3′
	JAG1-revE4	5′- GCTTCAGCGTCTGCCACTG -3′
*SNAI1*	SNAI1-ForE1	5′- CCAATCGGAAGCCTAACTAC -3′
	SNAI1- RevE2.1	5′- CGGTGGGGTTGAGGATCTC -3′
*SPARC*	SPARC-For	5′- CTCTTTAACCCTCCCCTTCG -3′
	SPARC-Rev	5′- ATGGGCAAAGCTACAAATGG -3′
18S RNA	18S-for	5′- CGCGGTTCTATTTTGTTGGT -3′
	18S-rev	5′- ATGCCAGAGTCTCGTTCGTT -3′
**Primers Used for Promoter Amplification**		
**Primer**	**Primer Sequence**	
FAP-for_2145	5′- CCTCCCTAAACCATGAATTC -3′	
FAP-NcoI-Rev	5′- TACCATGGTCTGATCACGTTCAATCCAG -3′	
FAP-HindIII-for	5′- TAAAGCTTTCTAGCCTGTGCATACACAC -3′	
CXCL12-HindIII-for	5′- CGCAAGCTTCATCTAACGGCCAAAGTGGT -3′	
CXCL12-NcoI-Rev	5′- TTCCATGGTGGCCAGCACGACCACGACCTTG -3′	
SDF1-F630	5′- GGAAACTGAGGCTCGGCTGGT -3′	
CTGF-HindIII-for	5′- AATAAGCTTGTGGACAGAACAGGGCAAAC -3′	
CTGF-NcoI-Rev	5′- TTCCATGGGTCGCACTGGCTGTCTCCT -3′	
JAG1-HindIII-for	5′- CACAAGCTTAACCGGCCGCTGAATAGTCA -3′	
JAG1-NcoI-Rev	5′- TTCCATGGTGGTCCGTGGGGAACGCATCG -3′	
SPARC-F2-For	5′- GATTGTGGCATGTGCGCCTGT-3′	
SPARC-NcoI-Rev	5′- CCATGGACCTCAGTGGCAGGCAGG-3′	

## Data Availability

The data presented in this study are available within the article text, figures, and Supplementary Materials.

## Supplementary Materials

The following are available online at https://www.mdpi.com/1422-0067/22/7/3298/s1, Supplementary S1: Nucleotide sequences of promoters used for this study.
