# Supplementary material for: Possibility for Transcriptional Targeting of Cancer-Associated Fibroblasts—Limitations and Opportunities
Source: Int J Mol Sci. 2021 Mar 24;22(7):3298. doi: 10.3390/ijms22073298 (PMC8038081; doi:10.3390/ijms22073298)
Supplement: Supplementary file 1 [file ijms-22-03298-s001.pdf]

**Nucleotide sequences of promoters used for this study:**

**>FAP 2.2 (2144bp)**

cctccctaaaccatgaattcagatggaaaaactcgacatctttatttctgcagtcagtctcatttttctt
aaaacagttcaaactagtaagaattttccagaagttacagcttgactcacccaaccttccaaggaaaaaa
caaaaaaacttaaacagacattgtttcactctcatcatttcccacccttactaatagtggcaacttaagt
gtatcttaaagcactccaacctcttcatagagcctattaaatgagtatcttgtggacacccacacacagt
catagaatcctaagtggtgctcagaccagtcacatgtcagtgcattcttaattgctagagctaacatgct
ctcagcatggtcttttaattacaccctaataatttattatagtttctctctacaatgtaaagtcttggaa
atcacccactaaaaagtgcctgtgtactctggggctttggcaggctagggcagaacttctgagaacacgg
tgtgttccagagaagacaatcaatctgagaggacttacacagaaacagttcattcaggacctggctgctg
gcttttatctgagatctgaggatttcacaatcacttggagatacctacaagtgtatagcacaccttggat
attactcttaatgattacttcattttgtaaagaggtgactccaccaacagcaaaggagagggcccagccc
cagccaccaggaatacagttctctgccagtaagtgcctaatgactcatttcctcaacagaattttcata
aggctggaattcagggagggatgtctggagaatgtctgaaaggaagttcacaagccactgtcctgctctt
tgctggagaaagtgtcccgtggtagccagagaagttgactaaggcaaacagcaacatgttttggtaacat
ttccccattacctttcatgtacaatccaagaaaggttgccatgaagtgttttaatcaggttgggaacatt
ataaacttcgaaaaaagaaaaccattagtggaaaaattaaggacacagtagatttaacaactgtgtttac
gtggaaccacaaaatctatccaagtgaattgcattaaaacagacagaacactccaagaaactgttgtatg
tgtatttttttaattcagtcaaccattttactaatctgtcaagatgaccaatttctttggaattatgta
gatttagccaaaatgaaattatacataagatttacttttcttttcagatgctttttatttattttaaa
tctttataattactagatgttctcctctctcagaagatattctgagaggaaagcaaaaataccactcttg
taaagccatttccattcttccaaaggtctgctggtaaattattcttactgatctttccatctttctagcc
tgtgcatacacacctaacccatactaaatttcaccagatggcattttatttctttaaagtaaagcagccg
tgggtttagacagttgaatttttaaacttctgtatttactgaaagtgcatatggtgctatatggacaaag
aaattgtgctgaaagaaaaacatttctgtctgcaatacctcataatcttccagaggaaaaaaaagtgcag
ttatatggcacatttctcacaaaatcttatgtggcttcaatgttcttcctctgttaaaaagtagatatat
gtttaatgtacagacctgcaagtttcattattttaaattcatcttttagtggcaaataaaaatgttatgc
aaaacccaatgacttgctaaagtgatccttcagtgaattctagaagaaaatgcaacataaacctgaactg
gtaaaaaagaaaaaataaaaacctctgtatgtcaacgtaagcagatgttggtgtagttacaaggatgaga
aggctataaaacttcccttgagtcactcacagttcatttgagggccaagaacgcccccaaaatctgtttc
taattttacagaaatcttttgaaacttggcacggtattcaaaagtccgtggaaagaaaaaaaccttgtcc
tggcttcagcttccaactacaaagacagacttggtccttttcaacggttttcacagatccagtgacccac
gctctgaagacagaattagctaactttcaaaaacatctggaaaa

**>FAP 0.75 (750bp)**

ctagcctgtgcatacacacctaacccatactaaatttcaccagatggcattttatttctttaaagtaaag
cagccgtgggtttagacagttgaatttttaaacttctgtatttactgaaagtgcatatggtgctatatgg
acaaagaaattgtgctgaaagaaaaacatttctgtctgcaatacctcataatcttccagaggaaaaaaaa
gtgcagttatatggcacatttctcacaaaatcttatgtggcttcaatgttcttcctctgttaaaaagtag
atatatgtttaatgtacagacctgcaagtttcattattttaaattcatcttttagtggcaaataaaaatg
ttatgcaaaacccaatgacttgctaaagtgatccttcagtgaattctagaagaaaatgcaacataaacct
gaactggtaaaaaagaaaaaataaaaacctctgtatgtcaacgtaagcagatgttggtgtagttacaagg
atgagaaggctataaaacttcccttgagtcactcacagttcatttgagggccaagaacgcccccaaaatc
tgtttctaattttacagaaatcttttgaaacttggcacggtattcaaaagtccgtggaaagaaaaaaacc
ttgtcctggcttcagcttccaactacaaagacagacttggtccttttcaacggttttcacagatccagtg
acccacgctctgaagacagaattagctaactttcaaaaacatctggaaaa

**>CXCL12 1.5 (1517 bp)**

catctaacggccaaagtggttttggaaaaaaaatgcacagaagacacctactcccaccagcggagttccg
gagccctcgcagcctcctgttgaccgctcccgcctaatgcagccgctgaccgcccactccccgacggcca
ggactccccagggacagggacgtgtccccagggcaggcccctggatggacgcggcgactgaccccacttc
gctggacgctgtgctgggaaggacacagagaggtggctggggcagcctgcggtcacaaagcgaggcccaa
aggggcgctctcctcacccccacgtctcctgggtgccgacctgcaccctcccttcgccaccggactgggg
ccatctgggatgtctcgggggtatccggagggctaagcaccgccgagggacggctccgtgggaagagttt
tctggacccagaaggcagacgccagtagtactgtcctaggagtcggaggtcggggtgggggagttctcag
ctctttgggtcgcacggagcttttcttgggtaaggcagtaagtacttaggtttaaaggacttacttacag

ctaccatttattgagtactgtctgcttgtcagacacgatgcagagaatttcgcggcgtggggcgggtctc
attgaatctcccgtcccactccgcggggtgggcctgtgattagctcatttcaccattgagaggtcggaag
tacaaaggctacattcgcttttactgagagccgccggcgccttctgctttgtttgtacaggcgaggaaac
tgaggctcggctggtggcgccgtgggcttggagtccgagccacgctgactgcaaagacgggtctcattcc
cgcagatcgagctctgccggcggctgcgccgcaagccgggcaggtggcgagcttgagcccccacgcacag
aaagcaggacccccctcggctgccttgggccgccaccgccagcaggccctccgcccgggactaacttgttt
gcttttcattggttctcattcagttcccgccatcgaaaggccccgtcccgcagctttccacgcgcgcccc
actttacgcctaaggtcctcagtctctccagtggggccctgtcacagggacaataagcggccctccagcc
ggcgtcgctcaggctgcggacctcactgcagaccgggccagcggtgcggggcccagcggagcctgagaag
gtcaaaggccggagcgcactgcgcctcgggagcacagagggagcggaggaggggcgaaggggatgggtgg
ggggtgccgccgagggagtcgcgcgtcagagaccccggccacggccagcactcggctccgggcccgcccc
tcaccgcgcgccccgccccgccccgcctggctctcccctctaaagcgcccggcgcgcgcctcccaccgcc
gcactttcactctccgtcagccgcattgcccgctcggcgtccggcccccgacccgcgctcgtccgcccgc
ccgcccgcccgcccgcgccatgaacgccaaggtcgtggtcgtgctgg

**>CXCL12 0.7 (753 bp)**

Ggaaactgaggctcggctggtggcgccgtgggcttggagtccgagccacgctgactgcaaagacgggtct
cattcccgcagatcgagctctgccggcggctgcgccgcaagccgggcaggtggcgagcttgagcccccac
gcacagaaagcaggacccccctcggctgccttgggccgccaccgccagcaggccctccgcccgggactaac
ttgtttgcttttcattggttctcattcagttcccgccatcgaaaggccccgtcccgcagctttccacgcg
cgccccactttacgcctaaggtcctcagtctctccagtggggccctgtcacagggacaataagcggccct
ccagccggcgtcgctcaggctgcggacctcactgcagaccgggccagcggtgcggggcccagcggagcct
gagaaggtcaaaggccggagcgcactgcgcctcgggagcacagagggagcggaggaggggcgaaggggat
gggtggggggtgccgccgagggagtcgcgcgtcagagaccccggccacggccagcactcggctccgggcc
cgcccctcaccgcgcgccccgccccgccccgcctggctctcccctctaaagcgcccggcgcgcgcctccc
accgccgcactttcactctccgtcagccgcattgcccgctcggcgtccggcccccgacccgcgctcgtcc
gcccgcccgcccgcccgcccgcgccatgaacgccaaggtcgtggtcgtgctgg

**>IGFBP2 (634bp)**

Ctagacgggtctgaaactccgcaggacccacccaacaagaagtcattgttccaagccacgtgtcagtggt
gggtgataccccaggatggaaggagttggtatgagccgactgaaatctacttgaaggtcaaaacggagcc
ttatgtcttttgtgttccccagcggttagcccagtgccggccacaggggaagcgcgcaaacgaagtcctc
gcgaactgaactgagagcagacaaaagcacgcgctcttctccaccgccacgccggtcctacccaaacccg
cgagttatccgtattctccttcaggagtcatagtcaggccagaagagtgcggagggacggggcccgggaa
gagcagggaacccccagagcccgcagccaacgcggaggtgggcgagcgggcgtgcgcgcactcacttgcc
ggcgcgagggagtgtcggggggggaagggagtggtctccaaaaggggggaggggagaaggcagggggcgggg
agaagccggccctttaggacccggctgcggcggcgagggaggaggaagaagcggaggaggcggctcccgc
gctcgcagggccgtgccacctgcccgcccgcccgctcgctcgctcgcccgccgcgccgcgctgccgaccg
ccag

**>CTGF (408bp)**

Gtggacagaacagggcaaacttattcgaaaaagaaataagaaataattgccagtgtgtttataaatgata
tgaatcaggagtggtgcgaagaggatagggaaaaaaaaattctatttggtgctggaaatactgcgctttt
ttttttcctttttttttttttctgtgagctggagtgtgccagctttttcagacggaggaatgctgagtgt
caaggggtcaggatcaatccggtgtgagttgatgaggcaggaaggtggggaggaatgcgaggaatgtccc
tgtttgtgtaggactccattcagctcattggcgagccgcggccgcccggagcgtataaaagcctcgggcc
gcccgccccaaactcacacaacaactcttccccgctgagaggagacagccagtgcgac

**>JAG1 (1724bp)**

aaccggccgctgaatagtcacgcttttctgcaggacatacctactattagggccaaaactttgtccaccc
ttcaaaggaagtcgatgtttccatataaaggtcccctcaaatgcaacagcaagcccgtgggagaggggtt
agcagaggctacggccagcccacgggctttcttcgaccttgattatgaccaggagtgtagctgttaattg
cgaggcttgcctcaaggtggaaaacagtatcggtttccactgccaccccagagaaggcaagttccgcggc
tggcggtgctggggacacggtccctcccaggcccatctcttgccacccagagagctgctcggaggccgcc
tacaggtgcaatcccggcactgcggccggggcgtcgggccggggagggcgtccaagcccaccagcatctc
cgccggcccttcccaaagcctgaacagggccccggcgtgcccgccgccttctacccccggtttccccgcg
cctctgccccggcgcggtttggataggaagctgggagccctcccaggctccgcagactcggatttgggag

ggggtgggacgcggccgaggcttcccctcgaatctgcggcaagcctggctccaggaaagtttttcaaagt
tcccagcagcgtctgcccaggtcgcctccgcggggcgagcagacggcggcaagcgcgccagcctcgccgc
cgcctctgccgccagcagagcgctctgggcggctcgctcgcgggaagcgggccgaactcccggcgggcag
gcaggccctcctcccggggcgaaagccgcagctgacgcaggcggttcggaaggcggaagctgccccgctc
cgaccgctcagtcagcgccgcggcgcctacacctggggccccgacgcgcgggcaaaggcgcacggcccgg
ggcgcccgagggggcggtccccgctgggggcctccaggcgtccctgagcaacgatcccttccaagtacct
ccccgcactctcccttccctcctggcccgaagctcccgagggcgggggttggtgtggggccctggttctt
ctacgccgccctgagcatcccgctgcccccaaccccttccaagttcctcctcgcactacccccctccccag
caacgtgaaggggaggggcgtgcccagggtgagcacgccctctcatgaatattaataagcgcgcatgcgc
cctgcccggcgtgctgggtagaggtggccagccccggccgctgctgccagacgggctctccgggtccttc
tccgagagccgggcgggcacgcgtcattgtgttacctgcggccggcccgcgagctaggctggttttttt
tttctcccctccctccccccttttccatgcagctgatctaaaagggaataaaaggctgcgcataatcat
aataataaaagaaggggagcgcgagagaaggaaagaaagccgggaggtggaagaggagggggagcgtctc
aaagaagcgatcagaataataaaaggaggccgggctctttgccttctggaacggccgctcttgaaaggg
cttttgaaaagtggtgttgttttccagtcgtgcatgctccaatcggcggagtatattagagccgggacgc
ggcggccgcaggggcagcggcgacggcagcaccggcggcagcaccagcgcgaacagcagcggcggcgtcc
cgagtgcccgcggcgcgcggcgcagcgatgcgttccccacggac

**>SNAI1 (929 bp)**

gataattcttcacttcctctgggaagtcaccccgacccccctgtcaggtgacccgcctcttaacggtcgcc
gcgtcccgtctctcccccaccaaagcacacttccctttgcattgtaattatctgtttacttcgtctgtct
ccctcactggaccagaagctacccttcgggagaggctctgagtgttctgtccggggctgtgccctggccc
caggtacagtgccccacacgtgctgggcgctccgtaaacactggataagggaaggaacgggtgctcttgg
ctagctgggccaggctgctttgcaaaaaggccgtggcatttcaagccgccgagagccacgtgcggtgtcc
ctttcctcgcttcctccccagtgatgtgcgtttccctcgtcaatgccacgctctccaggcgccagccggg
cggaggaaatttccgcccccctcccaagcccgaggcgggggcgggcgtcggaaggtcaggtgtcccggccg
gcgcgcagcgccaggggggcgtcagaagcgctcagaccaccgggcgctgagccggtgggcgcgcggcgtcc
tgccggggtcccacctcgcagaggcctcgcttcgctcgacgtcccgccccggacagccccagcaccgggg
acgacccgcgctgcgccagcgaaccccgcctcggaggagtccccgcccgggctctcaccgccacgcggcg
cgagcccggccagcagccggcgcacctgctcggggagtggccttcggcggagacgagcctccgattggcg
cggaggtgacaaaggggcgtggcagataaggccccgcccctcccacccccccaccaccccccccggagtact
taagggagttggcggcgctgctgcattcattgcgccgcggcacggcctagcgagtggttcttctgcgcta
ctgctgcgcgaatcggcga

>**SPARC (1234bp)**

tggcatgtgcgcctgtaatcccagctactctggaggctgaggcgcgataattgcttgaacccgggaggca
gaggttgcagtgagccgaaatcataccactgcactccagcctgggcgacagagtgagtgagactctgtct
caaaacaaaacaaaacaaacaaacaaaaaaaaccggaaaccacaaaactttttgaggacaaggaccaggta
tttattaattctcatacctcccagagtgttaggcacaaaataaacattcaaccaagacctgttgcactga
gcagttcatatataacaggagtgacccaagttgaaacgtagaatcagccctctcataccactttttgcca
ggtgatcataggcaagttacttagcatctatgtttccttattattaaaatggtcataattacaatgccta
agataaggggttgctgtgaagattattaaatcctcagtaaactttggctattgttactcctatgattatc
atcaatatcatcaattaccttatctgttcaatactggtggcacaggtccaccagctagatgtctaatccc
ttatgtgtctattagtggtacaagtggagtttgagtgggattttttttttaagaccagttccaaatcat
caaggatgataccactagtagcagcttgtcttgtctgtacagtggtaagtcctggccttgcctttgtggc
aaatacaaccccccttgaattgcttggcccttctcagcattgcctaatattagggaggactcctgtaaagc
tcactggttagaagatcaagacacttgggcctggttctgccccctgggggccattgggtaattccttgcag
tctccaggcctcacttgccctctgaacaagaaagaggctgttctgggtcatccctccaggcctgtccagc
cctggcactctgtgagtcggtttaggcagcagccccggaacagatgaggcaggcagggttgggacgtttg
gtcaggacagcccaccgcaaaaagaggaggaaagaaatgaaagacagagacagctttggctatgggagaa
ggaggaggccgggggaaggaggagacaggaggaggagggaccacggggtggaggggagatagacccagcc
cagagctctgagtggtttcctgttgcctgtctctaaacccctccacattcccgcggtccttcagactgcc
cggagagcgcgctctgcctgccgcctgcctgcctgccactgagg
